# A cross-sectional study of SARS-CoV-2 antibodies among healthcare workers in a tertiary care hospital in Taiwan: implications for protection against the Omicron variants

**DOI:** 10.1186/s12879-024-09411-z

**Published:** 2024-05-27

**Authors:** Chang-Hua Chen, Day-Yu Chao, Chew-Teng Kor, Su-Feng Kuo, Jen-Shiou Lin, Huei-Wen Lai, Yen-Tze Liu, Ching-Hsiung Lin, Mu-Kuan Chen

**Affiliations:** 1 Division of Infectious Diseases, Department of Internal Medicine, Changhua Christian Hospital, Changhua; No.135, Nanxiao St., Changhua City, Changhua County 50006 Taiwan; 2 Department of Post-Baccalaureate Medicine, College of Medicine, National Chung-Hsing University, No. 145 Xingda Rd., South Dist, Taichung City, 40227 Taiwan; 3 Doctoral Program in Microbial Genomics, National Chung Hsing University and Academia Sinica, No. 145 Xingda Rd., South Dist., Taichung City, 40227 Taiwan; 4 Graduate Institute of Microbiology and Public Health, College of Veterinary Medicine, National Chung-Hsing University, No. 145 Xingda Rd., South Dist, Taichung City, 40227 Taiwan; 5 Big Data Center, Changhua Christian Hospital, No.135, Nanxiao St., Changhua City, Changhua County 50006 Taiwan; 6 Institute of Statistics and Information Science, National Changhua University of Education, Changhua County, No.1, Jinde Rd, Changhua City, Changhua County 50074 Taiwan; 7 Clinical Microbiology Laboratory, Changhua Christian Hospital, No.135, Nanxiao St., Changhua City, Changhua County 50006 Taiwan; 8 Center for Infection Prevention and Control, Changhua Christian Hospital, No.135, Nanxiao St., Changhua City, Changhua County 50006 Taiwan; 9 Department of Family Medicine, Changhua Christian Hospital, No.135, Nanxiao St., Changhua City, Changhua County 50006 Taiwan; 10 Division of Chest Medicine, Department of Internal Medicine, Changhua Christian Hospital, No.135, Nanxiao St., Changhua City, Changhua County 50006 Taiwan; 11 Department Of Otorhinolaryngology - Head & Neck Surgery, Changhua Christian Hospital, No.135, Nanxiao St., Changhua City, Changhua County 50006 Taiwan

**Keywords:** COVID-19, Healthcare workers, Serology, SARS-CoV-2, Omicron variant

## Abstract

**Background:**

Taiwan, deeply impacted by the 2003 SARS outbreak, promptly implemented rigorous infection control and prevention (ICP) measures in January 2020 to combat the global COVID-19 pandemic. This cross-sectional serologic study was conducted among healthcare workers (HCWs) in a tertiary care hospital in Taiwan from August 1, 2022, to February 28, 2023. The study aimed to assess HCWs’ antibody responses to COVID-19 vaccination against Omicron subvariants BA.1, BA.4, and BA.5, considering variations in prior infection. Additionally, it evaluated the effectiveness of ICP and vaccination policies within the hospital setting in Taiwan.

**Methods:**

A cross-sectional serology study was conducted in Taiwan to investigate the seroprevalence rates of Omicron subvariants BA.1, BA.4, and BA.5 among HCWs. A total of 777 HCWs participated in this study. A structured questionnaire was collected to obtain the epidemiological characteristics and risk factors for potential exposure. Enzyme-linked immunosorbent assay was used to detect antibody responses. Serum samples were selected for protection against Omicron subvariants BA.1, BA.4, and BA.5 by using a pseudotyped-based neutralization assay.

**Results:**

More than 99% of the participants had received SARS-CoV-2 vaccination. Overall, 57.7% had been infected with SARS-CoV-2, with some being asymptomatic. The SARS-CoV-2 Anti-Spike S1 protein IgG (Anti-S) distribution was 40,000 AU/mL for 20.2% (157/777) of participants, with a mean ± standard deviation of 23,442 ± 22,086. The decay curve for Anti-S was less than 20,000 AU/ml after 120 days. The probability curve of 50% neutralization showed an Anti-S of 55,000 AU/ml. The optimum Anti-S was 41,328 AU/mL (equal to 5,869 WHO’s standard BAU/mL), with 86.1% sensitivity and 63.5% specificity.

**Conclusions:**

In this significant study, 20.2% of HCWs achieved seroprotection against Omicron subvariants BA.1, BA.4, and BA.5. Their immunity against Omicron subvariants was further reinforced through recommended vaccinations and the development of natural immunity from SARS-CoV-2 exposure, collectively enhancing their protection against Omicron.

**Supplementary Information:**

The online version contains supplementary material available at 10.1186/s12879-024-09411-z.

## Background

Since the onset of coronavirus disease 2019 (COVID-19) pandemic in late 2019, healthcare workers (HCWs) have been on the frontlines to combat this menace. In the last 28-day period (31 July to 27 August 2023), more than 1.4 million new COVID-19 cases and over 1800 deaths were reported by the World Health Organization, an increase of 38% and a decrease of 50%, respectively, compared with the previous 28 days [1]. Logistic, personnel [2], and infection control measures have been implemented in hospital settings to protect HCWs and prevent COVID-19 transmission among healthcare facilities [3]. The protection conferred by past infection from pre-Omicron variants against re-infection was very high and remained high even after 40 weeks [4]. However, protection was low for the Omicron subvariants and decreased rapidly over time [4]. A hypothesis of hybrid immunity was proposed, and both mixed vaccine-derived and natural population level immunity were complex [5, 6]. How the vaccination under different prior exposure history provide protective immunity against ongoing circulating Omicron variants remains unknown.

Globally, each country has developed specific strategies to defend against the COVID-19 pandemic [7]. . Taiwan was one of the countries heavily affected by the severe acute respiratory syndrome (SARS) outbreak in 2003. Therefore, since January 2020, Taiwan has implemented relevant strategies of infection control and prevention (ICP) measures, including surveillance, allocation of personal protective equipment (PPE), and COVID-19 advance education [8]. In Taiwan, the main policies of ICP measures include quarantine, rolling definition for case reporting and detection, contact tracing, wearing of surgical mask, social distancing, and isolation of patients with COVID-19 [9]. Taiwan also integrated severe acute respiratory syndrome coronavirus 2 (SARS-CoV-2) genomic surveillance into COVID-19 surveillance to detect variants of concern (VOCs). According to viral surveillance data, various variants emerged between December 2019 and March 2023, as illustrated in Supplement Fig. 1. Importantly, Before and after the ICP policies were relaxed, the seroprevalence was 0.05% [10], which suggested the majority of the population including the HCWs are not exposed to virus and the only protective immunity is from the vaccination. Therefore, a cross-sectional seroprevalence study was performed among HCWs in a tertiary care hospital in Taiwan from August 1, 2022, to January 1, 2023. We aimed to (1) evaluate the antibody response mainly from COVID-19 vaccination against Omicron subvariants BA.1, BA.4, and BA.5 without any prior infection; (2) the effectiveness of ICP measures and vaccination policies and their implementation in hospital settings in Taiwan.

## Materials and methods

### Settings

The people in the rural areas of central Taiwan are mainly served by the Changhua Christian Hospital System (CCHS), which has a 4000-bed capacity. Changhua Christian Hospital (CCH) is a 1654-bed tertiary referral medical center located in central Taiwan and is the largest hospital among the nine branches of the CCHS. On August 2023, a total of 4472 HCWs were working in CCH. In 2022, 3926 patients with COVID-19 were hospitalized at CCH. On March 20, 2023, there were 112 patients with COVID-19 who were hospitalized at CCH. The first patient with COVID-19 was admitted on January 21, 2020; to date, patients with COVID-19 are still being admitted to CCH. A 70-bed ward has been used as a COVID-19 quarantine ward since January 22, 2020.

The PPE regulations in CCH are implemented in the entire hospital and involve the use of an N95 mask, a face shield, a hair cover, an isolation gown, and gloves while caring for patients or performing oropharyngeal/nasopharyngeal swabs; these regulations were based on suggestions from the Centers for Disease Control of Taiwan [11].

### Participants

The HCWs of CCH who worked in the COVID-19 ward, provided care for suspected and/or confirmed patients with COVID-19, or considered themselves to have been exposed to COVID-19 were encouraged to participate in this study. An open e-mail invitation was sent to each HCW. The study protocol was reviewed and approved by the Research Ethics Committee of CCH (approval no. 221,012).

### Study design

This cross-sectional study was conducted from August 1, 2022, to February 28, 2023. After obtaining informed consent from the participants, we asked them to complete a structured self-administered questionnaire, which included questions on age, sex, underlying diseases, community-associated risk factors, and possible symptoms associated with COVID-19 (such as fever, cough) [12, 13]. We defined participants who were never positive for COVID-19 as follows: (1) self-reporting be cross-referenced with medical records, plus (2) participants undergo serological testing to confirm their current and past infection status. Hospital-associated risk factors were defined as working in the Center for Infection Prevention and Control, including the COVID-19 ward; close contact with patients with COVID-19 (< 1.5 m); or performing oropharyngeal/nasopharyngeal swab sampling for patients suspected of or positive for COVID-19.

### Evaluation of SARS-CoV-2 antibodies

Blood samples were collected once. Enzyme-linked immunosorbent assay (ELISA) was performed using the Architect SARS-CoV-2 IgG and IgG II Quant assay (Abbott-NP, Abbott, Chicago, IL) were used to detect antibody responses against SARS-CoV-2 [14–16]. The cutoff value for a positive IgG response was anti-Spike S1 protein IgG (Anti-S) < 50.0 AU/mL and anti-nucleocapsid IgG index (Anti-N) ≥ 1.4 according to the instructions of the manufacturer [14–16]. We have proceeded to convert the antibody titer measurements from arbitrary units per milliliter (AU/mL) to WHO’s standard BAU/mL, with BAU/mL being calculated as 0.142 times AU/mL.

### Pseudotyped-based virus neutralization assay

We conducted stratified random sampling from four classes, including Anti-S (> 40,000 AU/mL) + Anti-N (negative), Anti-S (40,000–20,000AU/mL) + Anti-N (negative), Anti-S (< 20,000 AU/mL) + Anti-N (negative), and Anti-S (> 40,000 AU/mL) + Anti-N (positive), with 25 samples selected from each stratum.

The serum samples screened by double-antigen binding-assay ELISA that detected antibodies recognizing the receptor binding domain of the SARS-CoV-2 S protein were selected for protection against Omicron subvariants by using pseudotyped-based neutralization assay. SARS-CoV-2 pseudotyped lentiviruses, which express full-length S proteins from Omicron subvariants BA.1, BA.4, and BA.5, and green fluorescent protein were used to infect HEK293T cells that overexpress human angiotensin I converting enzyme 2 (HEK293T/hACE2 cells) at a multiplicity of infection of 0.5. Both were purchased from the National RNAi Core Facility (Academia Sinica, Taiwan). The serum was diluted 500-fold in Dulbecco’s Modified Eagle’s Medium, followed by pre-incubation with a SARS-CoV-2 pseudovirus with a designated titer for 1 h at 37 °C. After incubation, the mixture was added to a 96-well black plate that was pre-seeded with 1 × 10^4^ HEK293T/hACE2 cells for 48 h at 37 °C. The intensity of GFP expression was measured by a spectrometer (FLUOstar OPTIMA, BMG, The Netherlands). The fluorescent intensity of GFP from the wells of the pseudovirus and cell culture medium were used as positive and negative controls, respectively. The relative percentage of neutralization (% neutralization) was calculated as follows: 100 × [1 - (GFP intensity of sample #/GFP intensity of pseudovirus only)]. The % neutralization of each sample was determined on the basis of three independent experiments.

### Statistical analysis

Categorical data are presented as percentages and numbers, and continuous variables are presented as mean + standard deviation (SD) or median with interquartile range when continuous data were not normally distributed. A confusion matrix was used to calculate the percentage agreement of the classification results of SARS-CoV-2 Anti-N with diagnosed COVID-19. We used logistic regression models to assess the relationship between the neutralizing levels of antibodies collected from participants and protection efficacy against COVID-19. Furthermore, we presented receiver operating characteristic analysis to determine the protective threshold established to maximize the area under the curve. We modeled quantitative antibody titers by day from last vaccination to test day by using generalized additive models after adjusting for age, gender, risk scale, working department, and total vaccination dose. We plotted Kaplan–Meier curves to estimate the cumulative incidence of the decrease in Anti-N antibody to a negative value and used the log-rank test to compare vaccination durations across groups. Two tailed p-value < 0.05 was considered significant. All analyses were performed with using SPSS (version 22.0 IBM Corp., Armonk, NY, USA) and R language (4.2.3 version) with “mgcv” and “splines” package.

## Results

### Demography

A total of 777 HCWs participated in this cross-sectional study (Table 1). The mean age was 39.0 ± 9.7 years, and 110 of the participants were men (110/777 [14.2%]). Most of the participants (87.8%) did not have underlying diseases. Among the participants, 4 (0.5%) had diabetes mellitus, 7 (0.9%) had chronic kidney disease, 15 (1.9%) had autoimmune diseases, and 15 (1.9%) had a history of cancer. Nineteen participants had more than one risk factor.

**Table 1:** Comparison between infected group and non-infected group in current study

	Total	Diagnosed with COVID-19		*P*-value
		No	Yes	
Sample size	777	353	424	
Gender				
Female	667 (85.8%)	297 (84.1%)	370 (87.3%)	0.213
Male	110 (14.2%)	56 (15.9%)	54 (12.7%)	
Age	39.0 ± 9.7	40 ±10	38 ± 9	0.061
Risk scale				
Suage of steroid	49 (6.3%)	19 (5.4%)	30 (7.1%)	0.334
Immunosuppressants	15 (1.9%)	7 (2%)	8 (1.9%)	0.923
Cancer	15 (1.9%)	5 (1.4%)	10 (2.4%)	0.342
Diabetes mellitus	4 (0.5%)	3 (0.8%)	1 (0.2%)	0.234
Kidney disease	7 (0.9%)	2 (0.6%)	5 (1.2%)	0.368
Cerebral vascular accident	17 (2.2%)	9 (2.5%)	8 (1.9%)	0.529
Tuberculosis	1 (0.1%)	0 (0%)	1 (0.2%)	0.361
Chronic Liver Disease	2 (0.3%)	2 (0.6%)	0 (0%)	0.121
Mental disorder	5 (0.6%)	1 (0.3%)	4 (0.9%)	0.252
Transplant by history	1 (0.1%)	0 (0%)	1 (0.2%)	0.361
Age ≥ 65	1 (0.1%)	0 (0%)	1 (0.2%)	0.361
Risk scale score				
0	682 (87.8%)	315 (89.2%)	367 (86.6%)	0.245
1	76 (9.8%)	28 (7.9%)	48 (11.3%)	
≥2	19 (2.4%)	10 (2.8%)	9 (2.1%)	
The number of vaccinations that participants have received				
Less two doses	4 (0.5%)	2 (0.6%)	2 (0.5%)	< 0.001
3 doses	183 (23.6%)	63 (17.8%)	120 (28.3%)	
4 doses	414 (53.3%)	162 (45.9%)	252 (59.4%)	
5 doses	176 (22.7%)	126 (35.7%)	50 (11.8%)	
Anti-N ratio(range)	0.38 (0.06, 1.53)	0.05 (0.02,0.17)	1.08 (0.45, 2.53)	< 0.001
Anti-N interpretation				
Negative	572 (73.6%)	329 (42.3%)	243 (31.3%)	< 0.001
Positive	205 (26.4%)	24 (3.1%)	181 (23.3%)	
Anti-S titer (AU/mL)				
Mean ± SD	23,442 ± 22.86	14,791 ± 18,798	30,644 ± 22,054	
Median (IQR)	15,993 (5632, 35,062)	6653.9 (2290, 18,250)	23561.8 (12,619, 47,975)	< 0.001
Anti-S titer > 40 K AU/mL	157 (20.2%)	34 (9.6%)	123 (29%)	< 0.001
Anti-S interpretation				
Positive	777 (100%)	353 (100%)	424 (100%)	1.00

Note: We defined participants who were never positive for COVID-19 as follows: (1) self-reporting be cross-referenced with medical records, plus (2) participants undergo serological testing to confirm their current and past infection status

More than 99% of the participants had received vaccination for SARS-CoV-2 (773/777 [99.5%]). Overall, 57.7% of participants had been infected by SARS-CoV-2 (Table 1), and some of them were asymptomatic for COVID-19. The distribution of the Anti-S of SARS-CoV-2 of 20.2% (157/777) of participants was more than 40,000 AU/mL, with a mean ± SD of 23,442 ± 22,086. The confusion matrix for the Anti-N of SARS-CoV-2 and the diagnosis of COVID-19 were analyzed, and the agreement value was 65.6% (Supplement Table 1).

### Probability curve between neutralizing antibodies and SARS-CoV-2 IgG

**Fig. 1:**
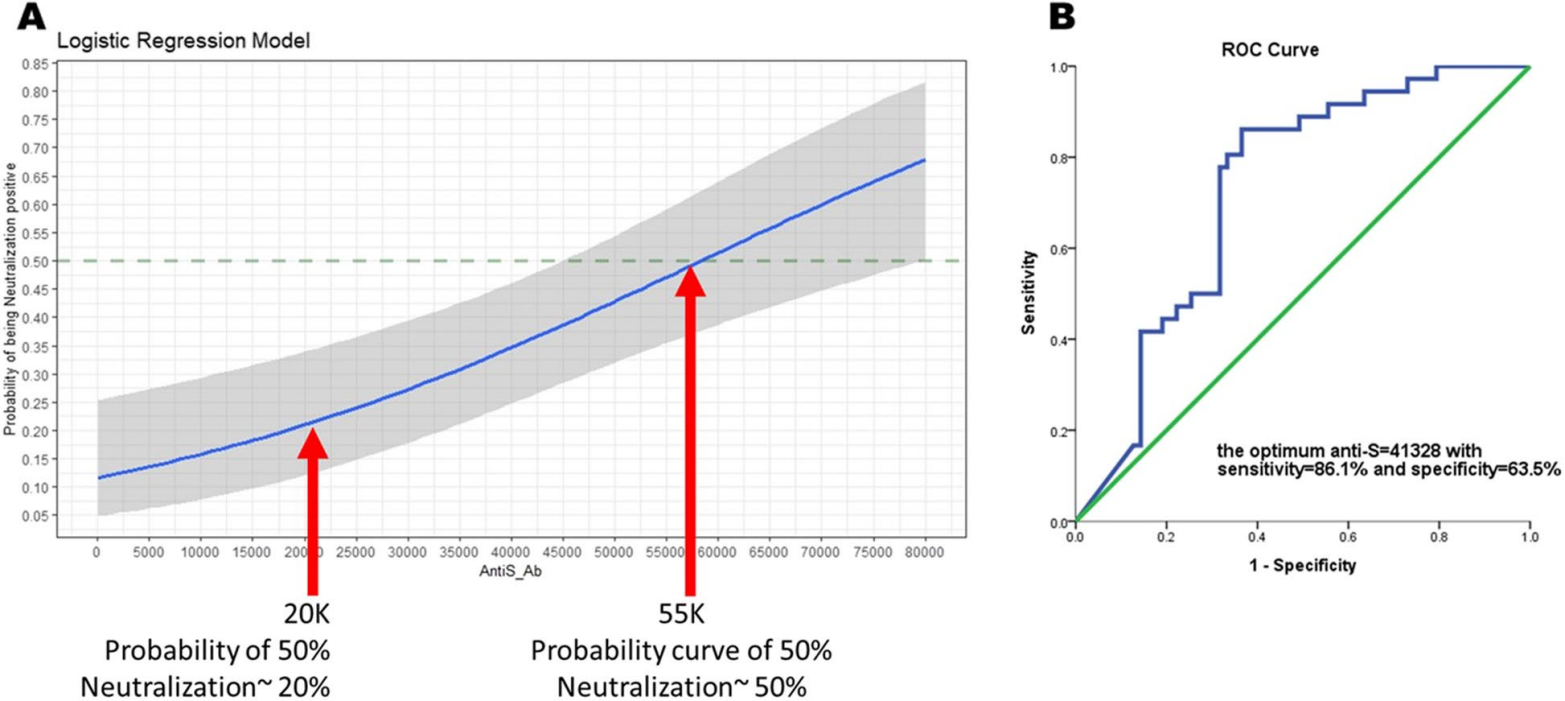
Probability curve between neutralizing antibodies and SARS-CoV-2 IgG titer. **A** The probability curve of Anti-S title with 95% confidence interval. **B** the optimal cutpoint of Anti-S based on ROC curve

We utilized a logistic model to determine the likelihood of achieving the protective neutralization across various levels of SARS-CoV-2 IgG. Our analysis revealed that SARS-CoV-2 IgG at 55,000 AU/mL corresponds to the estimated 50% protective neutralization threshold (Fig. 1). The optimal Anti-S was 41,328 AU/mL(equal to 5,869 WHO’s standard BAU/mL), with 86.1% sensitivity and 63.5% specificity (Fig. 1). The optimal Anti-S would achieve seroprotection against Omicron subvariants BA.1, BA.4, and BA.5.

### The decay curve for SARS-CoV-2 antibodies

**Fig. 2:**
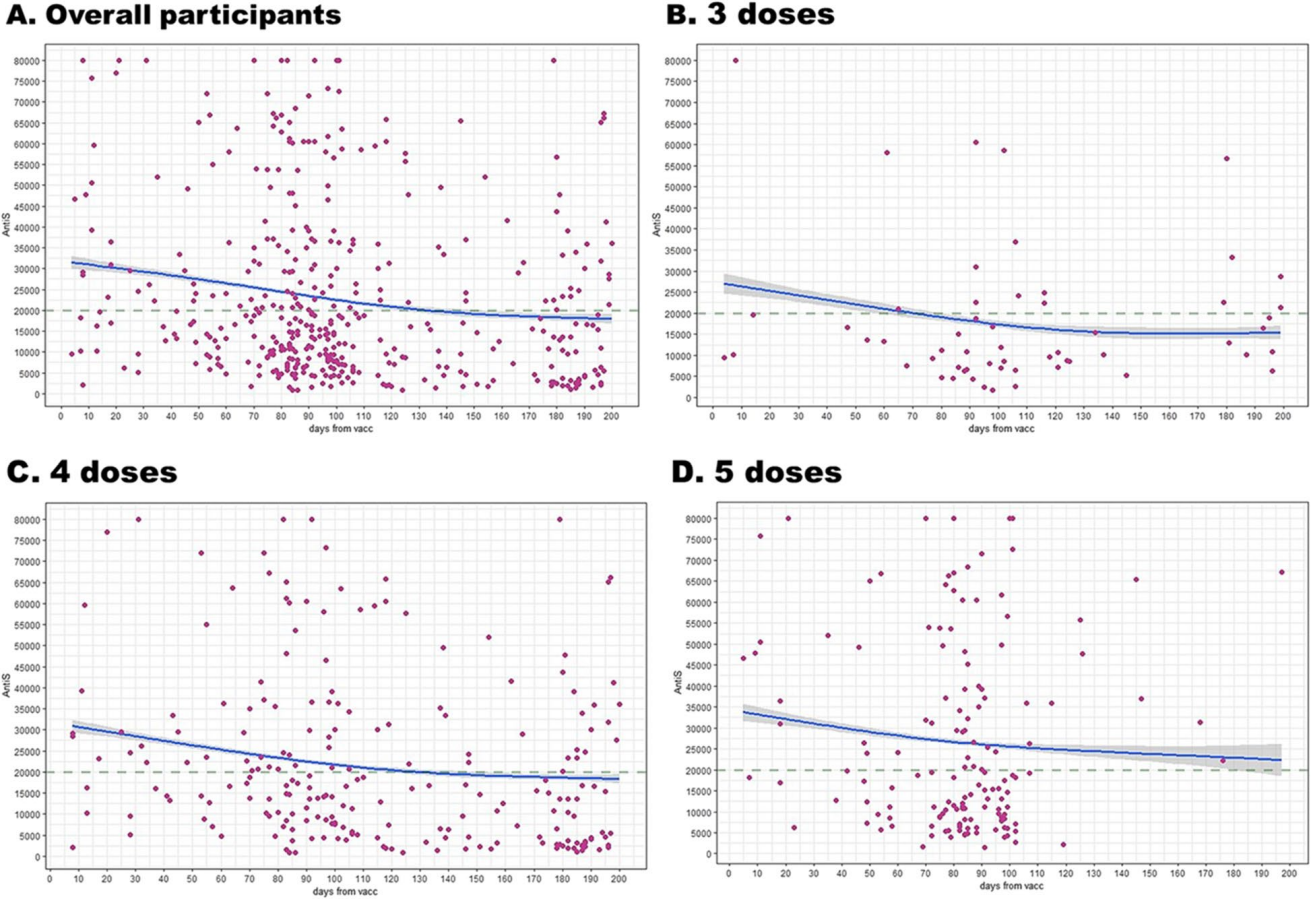
Anti-S titer decay curves after vaccination duration for all participants and stratified by vaccination dose

The Fig. 2 demonstrates the utilization of Generalized Additive Models to estimate the decay curve with varying numbers of shots. The decay curve of Anti-S indicates a decrease to less than 20,000 AU/mL after 120 days, with the rate of decay being influenced by the number of vaccinations.

**Fig. 3:**
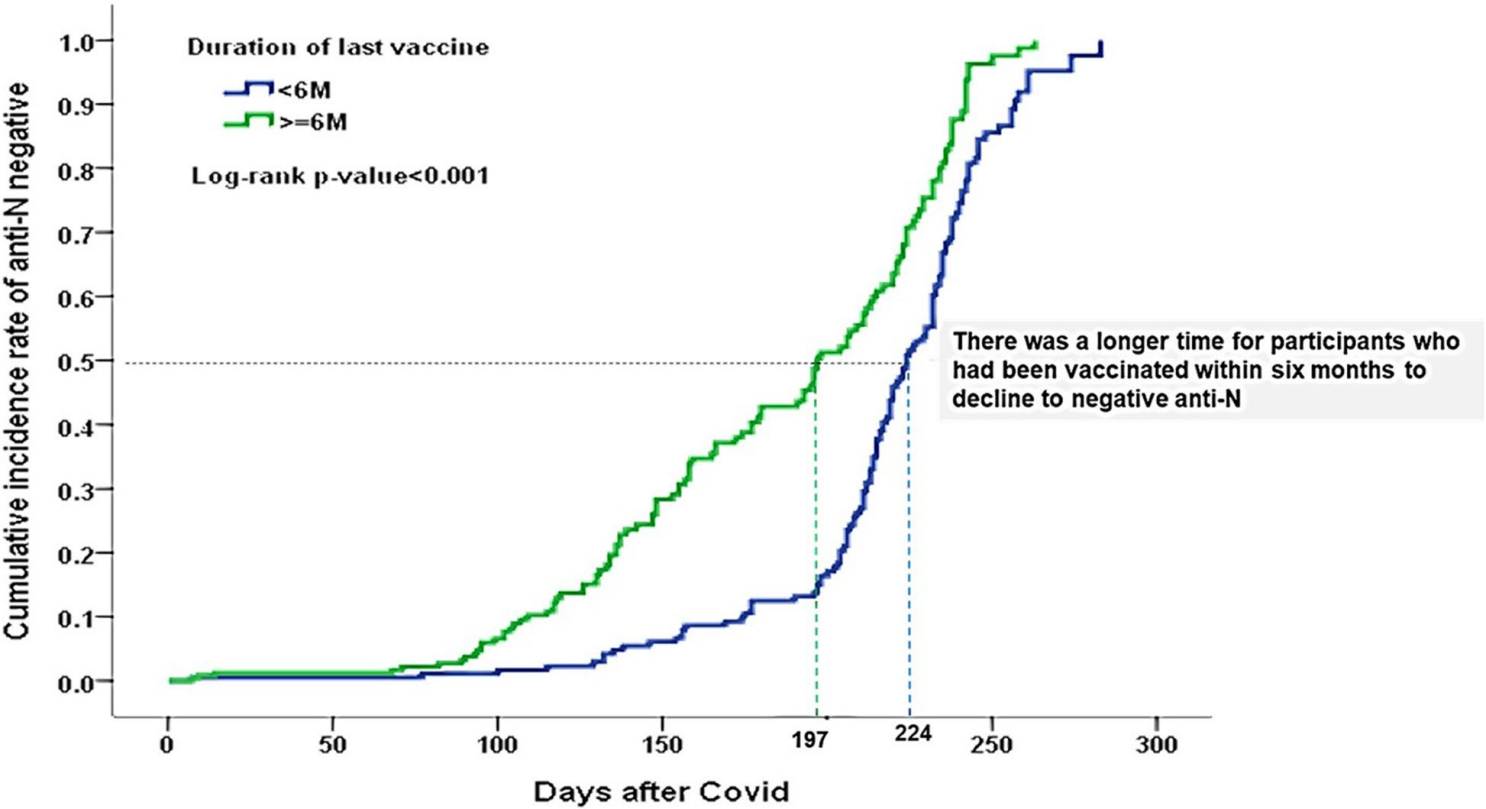
Cumulative incidence curve for the decrease in Anti-N to a negative value

Participants vaccinated within six months exhibited a prolonged duration to achieve negative anti-N titers. Figure 3 show the cumulative incidence curve towards negative anti-N titers.

### Distribution of SARS-CoV-2 antibodies

Both the Architect SARS-CoV-2 IgG and IgG II Quant (Abbott) were used for all participants. Each participant had a positive result in the Architect SARS-CoV-2 IgG test (Anti-S protein). The distribution of the Anti-S of SARS-CoV-2 for 20% of HCWs was more than 400,00 AU/mL, and the Anti-S titer among four units varied (Supplement Table 2). Participants were not mandated to receive vaccination, and we have provided a comprehensive breakdown of the types of vaccines administered (Supplement Table 3). Table 1 provides a comprehensive overview of the participants’ characteristics, including gender distribution, age, comorbidities, number of vaccinations, and antibody titers. Furthermore, to investigate the factors associated with COVID-19 infection, we conducted logistic regression analysis and summarized the results in Supplement Table 4. In Supplemental Table 4, we identified several significant factors associated with the risk of COVID-19 infection and calculated their respective odds ratios (OR) and 95% confidence intervals (CI). Specifically, our analysis revealed that a vaccination duration of less than 180 days (OR = 0.489, 95% CI [0.33,0.74], *P*-value = 0.001), an anti-S titer exceeding 40 K AU/mL (OR = 0.149, 95% CI [0.09,0.24], *P*-value < 0.001), and receiving more than 4 vaccination doses (OR = 0.584, 95% CI [0.39,0.88], *P*-value = 0.011 for 4 doses; OR = 0.068, 95% CI [0.04,0.13], *P*-value < 0.001 for 5 doses) were associated with a reduced risk of COVID-19 infection.

## Discussion

This cross-sectional study aimed to assess seroprotective immunity against the circulating Omicron subvariants BA.1, BA.4, and BA.5. The research unveiled a significant finding: 20.2% of HCWs had achieved seroprotection against these Omicron subvariants. Remarkably, our investigation further revealed that 20.2% of HCWs reached this threshold of neutralizing antibodies through vaccination, regardless of variations in prior exposure to COVID-19. Taking into account the four waves of infection in Taiwan and the vaccination policy, the observed outcomes can be attributed to the successful ICP implementation of Taiwan’s ICP measures within healthcare institutions, in conjunction with the concept of hybrid immunity [5, 6]. It’s important to emphasize that while Taiwan’s ICP policies have played a critical role in safeguarding HCWs from COVID-19 [9], the majority of protective immunity among HCWs was acquired through vaccination, subsequent to a relaxation of ICP measures. Notably, the Omicron subvariants offer only limited cross-protection [17]. As a result, in response to the Omicron subvariants emerging after the relaxation of ICP measures, HCWs received the recommended vaccinations. Some individuals also developed natural immunity following exposure to SARS-CoV-2. This multifaceted approach effectively generated a more comprehensive immune response, substantially increasing the likelihood that HCWs would attain sufficient protection against the Omicron variant.

Our findings in this study is similar to those of a previous report, and Omicron subvariants provided limited cross-protection [17]. In the current report (Figs. 2 and 3), the Anti-S and Anti-N antibody will decay gradually. Nowadays, the use of serology as a serosurveillance tool in the general population should be approached with caution because the specificity in the asymptomatic population has not yet been well documented [7].

Our report shares the perspective that establishing hybrid immunity, stemming from both vaccine-derived and natural population-level immunity, is a multifaceted endeavor [5, 6]. Zaballa et al. observed that less than 50% of individuals exhibit neutralizing activity against the circulating Omicron BA.5 subvariant following the Omicron BA.2 wave [18]. Achieving sufficient immunity poses a notable challenge, particularly for hospitals, which serve as primary recipients of new coronavirus cases. Some studies, targeting high-risk populations like HCWs, emphasize the importance of robust support and motivation from healthcare institutions [19]. Barrufet et al. found that the serologic distribution rate of SARS-CoV-2 antibodies among HCWs was twice that of the general population, with protective measures at work and in social settings associated with reduced infection risk, a trend stabilized post-vaccination [19]. Despite the limited number of individuals with adequate antibodies, Taiwan has effectively implemented ICP measures, evidenced by consistent COVID-19 infection rates across various hospital units during stringent ICP enforcement. The align with Chan et al.‘s study involving HCWs in Taiwan, where all nurses tested negative for SARS-CoV-2 [20]. Chan et al. reported that 195 HCWs were subjected to virological surveillance because of fever or any respiratory symptoms [20], and all nurses tested negative for SARS-CoV-2 [20]. This supports the efficacy of current ICP policies and adequacy of PPE regulations in Taiwanese hospitals. Moreover, Bryan et al. demonstrated the sensitivity and specificity of SARS-CoV-2 IgG, underscoring the reliability of serological testing [15]. Taiwan, having experienced the SARS outbreak in 2003, swiftly implemented relevant ICP strategies since January 2020, including surveillance, PPE allocation, and COVID-19 education [8, 21–24] (Supplemental Fig. 2). Stadler et al. highlighted the relationship between monoclonal antibody concentration and COVID-19 prevention efficacy, estimating that a concentration of 40,000 AU/mL provides a high likelihood of at least 50% protection against circulating Omicron subvariants BA.1, BA.4, and BA.5 [22]. Given the challenges posed by Omicron, only 20% of HCWs in our study attained the requisite neutralizing antibody levels through vaccination alone. Therefore, a combination of recommended vaccinations and natural immunity from SARS-CoV-2 infection offers a comprehensive immune response, enhancing HCWs’ chances of adequate protection against the ongoing Omicron variant.

The limitations of the study are mainly due to the nature of voluntary participation. Participants who want to participate in the study may be classified as more likely to be infected with COVID-19 because of their personal judgment. Therefore, this study tends to include a high-risk population and may underestimate or overestimate the serologic status of HCWs in the institution. Furthermore, the positive predictive value of the anti-SARS-CoV-2 antibody was low because Taiwan had a relatively high incidence of COVID-19 during the study period. However, if the duration of SARS-CoV-2 IgG is short, the serologic status is underestimated. He et al. conducted serology studies in different periods on nurses at Renmin Hospital at Wuhan University [25]. The time of IgG was long, and the second test showed that 71.8% of IgGs were seropositive. Paul et al. showed that Anti-N IgG has been a reliable marker of SARS-CoV-2 infection for more than a year [26]. However, in the unaffected population infected with COVID-19, there are no specific research objectives [27]. Additional studies are needed to clarify the average IgG survival period and to evaluate the seroprotection against from the newly emerging Omicron subvariant XBB. And, the re-infection rate of COVID-19 was only evaluated by self-report questionnaire, and the accuracy of the re-infection rate in the current study needs to be validated. Lastly, in our enrolled participants predominantly consisted of women and this could have affected the study results.

## Conclusion

In this fundamental cross-sectional study, 20.2% of HCWs achieved seroprotection against Omicron subvariants BA.1, BA.4, and BA.5, primarily through vaccination, irrespective of prior COVID-19 exposure. This protection was enhanced by Taiwan’s effective implementation of ICP measures and the concept of hybrid immunity. While ICP policies played a crucial role, vaccination post-ICP relaxation was the primary source of immunity. It’s important to note that Omicron subvariants offer limited cross-protection. Responding to the Omicron subvariants, HCWs actively received recommended vaccinations, complemented by some acquiring natural immunity from SARS-CoV-2 exposure, ultimately bolstering immunity against Omicron.

### Supplementary Information

Additional file 1: Supplement Table 1. The confusion matrix for Anti-N for SARS-CoV-2 and diagnosed with COVID-19. Supplement Table 2. The distribution of anti-S of SARS-CoV-2 among four different units. Supplement Table 3. The demography among different vaccine types. Supplement Table 4The logistic regression model to determine the association factors and COVID infection in current study. Supplement Figure 1. Timeline of different variants of concerns for SARS-CoV-2. Supplement Figure 2. Timeline of Taiwan’s COVID-19 infection prevention and control policies from 2021 to March 2023.

## Data Availability

In adherence to privacy regulations and institutional policies, the data underpinning this study are not publicly accessible. However, researchers seeking access to the data for verification or collaborative endeavors may contact the corresponding authors upon reasonable request. Such requests are subject to approval from the institutional review board of Changhua Christian Hospital and compliance with relevant data protection protocols.

## Abbreviations

Anti-N: Anti-nucleocapsid IgG index
Anti-S: Anti-Spike S1 protein IgG
CCH: Changhua Christian Hospital
CCHS: Changhua Christian Hospital System
CI: Confidence intervals
COVID-19: Coronavirus disease 2019
ELISA: Enzyme-linked immunosorbent assay
HCWs: Healthcare workers
ICP: Infection control and prevention
OR: Odds ratios
PPE: Personal protective equipment
RT-PCR: Reverse transcription polymerase chain reaction
SARS-CoV-2: Severe acute respiratory syndrome coronavirus 2
SD: Standard deviation
VOC: Variants of concern
