# Supplementary material for: A cross-sectional study of SARS-CoV-2 antibodies among healthcare workers in a tertiary care hospital in Taiwan: implications for protection against the Omicron variants
Source: BMC Infect Dis. 2024 May 27;24:529. doi: 10.1186/s12879-024-09411-z (PMC11129381; doi:10.1186/s12879-024-09411-z)
Supplement: Supplementary file 1 — Additional file 1: Supplement Table 1. The confusion matrix for Anti-N for SARS-CoV-2 and diagnosed with COVID-19. Supplement Table 2. The distribution of anti-S of SARS-CoV-2 among four different units. Supplement Table 3. The demography among different vaccine types. Supplement Table 4The logistic regression model to determine the association factors and COVID infection in current study. Supplement Figure 1. Timeline of different variants of concerns for SARS-CoV-2. Supplement Figure 2. Timeline of Taiwan’s COVID-19 infection prevention and control policies from 2021 to March 2023. [file 12879_2024_9411_MOESM1_ESM.docx]

List of supplementary information

Supplement Table 1

The confusion matrix for Anti-N for SARS-CoV-2 and diagnosed with COVID-19.

Supplement Table 2

The distribution of anti-S of SARS-CoV-2 among four different units

Supplement Table 3

The demography among different vaccine types

Supplement Table 4

The logistic regression model to determine the association factors and COVID infection in current study

Supplement Figure 1

Timeline of different variants of concerns for SARS-CoV-2

Supplement Figure 2

Timeline of Taiwan's COVID-19 infection prevention and control policies from 2021 to March 2023

Supplement Table 1

The confusion matrix for Anti-N for SARS-CoV-2 and diagnosed with COVID-19

|  | | Anti-N interpretation according to the Architect SARS-CoV-2 IgG and IgG II Quant assay | |
| --- | --- | --- | --- |
|  |  | Negative | Positive |
| Diagnosed with COVID-19 according to self-reports or medical records | No | 329  (42.3%) | 24  (3.1%) |
|  | Yes | 243  (31.3%) | 181  (23.3%) |

Note

Agreement percent : (329+181)/777 is 65.64%

Supplement Table 2

The distribution of anti-S of SARS-CoV-2 among four different units

|  | Officers of Hospital Incident Command System | COVID-19 dedicated unit | High Risk unit | non-High risk unit |
| --- | --- | --- | --- | --- |
| Distribution of anti-S titer | | | | |
| mean | 20025 | 14563 | 12315 | 16842 |
| range | (7165-55769) | (5123-32254) | (5121-36288) | (6307-35054) |
| <4K AU/mL | 5(9.8%) | 55(18.8%) | 21(19.8%) | 63(18.4%) |
| 4K -20K AU/mL | 20(39.2%) | 124(42.3%) | 46(43.4%) | 123(36.0%) |
| 20K-40K AU/mL | 8(15.7%) | 61(20.8%) | 15(14.2%) | 89(26.0%) |
| >40K AU/mL | 18(35.3%) | 53(18.1%) | 24(22.6%) | 67(19.6%) |
| The duration from the last vaccination shot to the timing of antibody measurement | | | | |
| Mean (range) | 190 days (88-214) | 203 days (97-356) | 173 days (84-260) | 182 days (89-211) |
| >6Month | 26 (51.0%) | 166 (56.8%) | 49 (46.2%) | 166 (56.8%) |

Supplement Table 3

The demography among different vaccine types

|  | BioNTech | Moderna_BA1 | Moderna_BA4/5 | Medigen | Moderna | Novavax | Uncertainty | P-value |
| --- | --- | --- | --- | --- | --- | --- | --- | --- |
| The number of vaccinations that participants have received | | | | | | | | |
| 3 | 14(21.2%) | 0(0%) | 0(0%) | 4(17.4%) | 125(51.9%) | 0(0%) | 40(20%) | <0.001 |
| 4 | 52(78.8%) | 70(40.9%) | 8(25%) | 19(82.6%) | 114(47.3%) | 43(97.7%) | 108(54%) |  |
| 5 | 0(0%) | 101(59.1%) | 24(75%) | 0(0%) | 0(0%) | 1(2.3%) | 50(25%) |  |
| Anti-N SC ratio (range) | 0.96 (0.11,2.44) | 0.25 (0.04,0.81) | 0.15 (0.05,1.27) | 0.54 (0.03,2) | 0.59 (0.09,1.93) | 0.27 (0.05,1.48) | 0.31 (0.06,1.29) | 0.003 |
| Anti-S titer (AU/mL) | 11530 (5589,27648) | 18696 (9611,37162) | 36689 (27944,52380) | 8012 (1510,22581) | 12949 (3325,31767) | 8228 (4258,29135) | 16813 (5312,36028) | <0.001 |
| Gender ,Female | 57(86.4%) | 142(83%) | 27(84.4%) | 21(91.3%) | 208(86.3%) | 39(88.6%) | 173(86.5%) | 0.907 |
| Age, mean (range) | 41(31,47) | 41(33,49) | 40(32,46) | 40(35,51) | 38(30,46) | 44(34,48) | 35(29,42) | <0.001 |
| The duration from the last vaccination shot to the timing of antibody measurement | | | | | | | | |
| Days(range) | 196(193,198) | 86(80,98) | 42(11,49) | 136(101,172) | 173(121,190) | 166(138,182) | 91(71,116) | <0.001 |
| Risk scale (including usage of steroid, usage of immunosuppressants, cancer, diabetes mellitus, kidney disease, cerebral vascular accident, tuberculosis, chronic liver disease, mental disorder, transplant by history) | | | | | | | | |
| 0 | 59(89.4%) | 146(85.4%) | 29(90.6%) | 21(91.3%) | 215(89.2%) | 33(75%) | 179(89.5%) | 0.031 |
| 1 | 6(9.1%) | 17(9.9%) | 2(6.3%) | 2(8.7%) | 18(7.5%) | 11(25%) | 20(10%) |  |
| ≥ 2 | 1(1.5%) | 8(4.7%) | 1(3.1%) | 0(0%) | 8(3.3%) | 0(0%) | 1(0.5%) |  |
| COVID19 | 45(68.2%) | 79(46.2%) | 14(43.8%) | 11(47.8%) | 144(59.8%) | 22(50%) | 109(54.5%) | 0.023 |

Supplement Table 4

The logistic regression model to determine the association factors and COVID infection in current study

|  | cOR(95% CI) | P-value | aOR(95% CI) | P-value |
| --- | --- | --- | --- | --- |
| age per 1-year increase | 0.986(0.97,1) | 0.060 | 0.99(0.97,1.01) | 0.250 |
| duration of vaccination < 180 days (range) | 1.31(0.99,1.74) | 0.059 | 0.489(0.33,0.74) | 0.001 |
| anti-S titer> 40K AU/mL (range) | 0.251  (0.17,0.38) | <0.001 | 0.149  (0.09,0.24) | <0.001 |
| Number of vaccination doses | | | | |
| 3 doses | 1 (reference) |  | 1( reference) |  |
| 4 doses(range) | 0.859(0.6,1.22) | 0.397 | 0.584(0.39,0.88) | 0.011 |
| 5 doses(range) | 0.218(0.14,0.34) | <0.001 | 0.068(0.04,0.13) | <0.001 |

Note:

Our analysis identified several significant factors, including a vaccination duration of less than 180 days, an anti-S titer exceeding 40K AU/mL, and receiving more than 4 vaccination doses, which were associated with a reduced risk of COVID-19 infection.


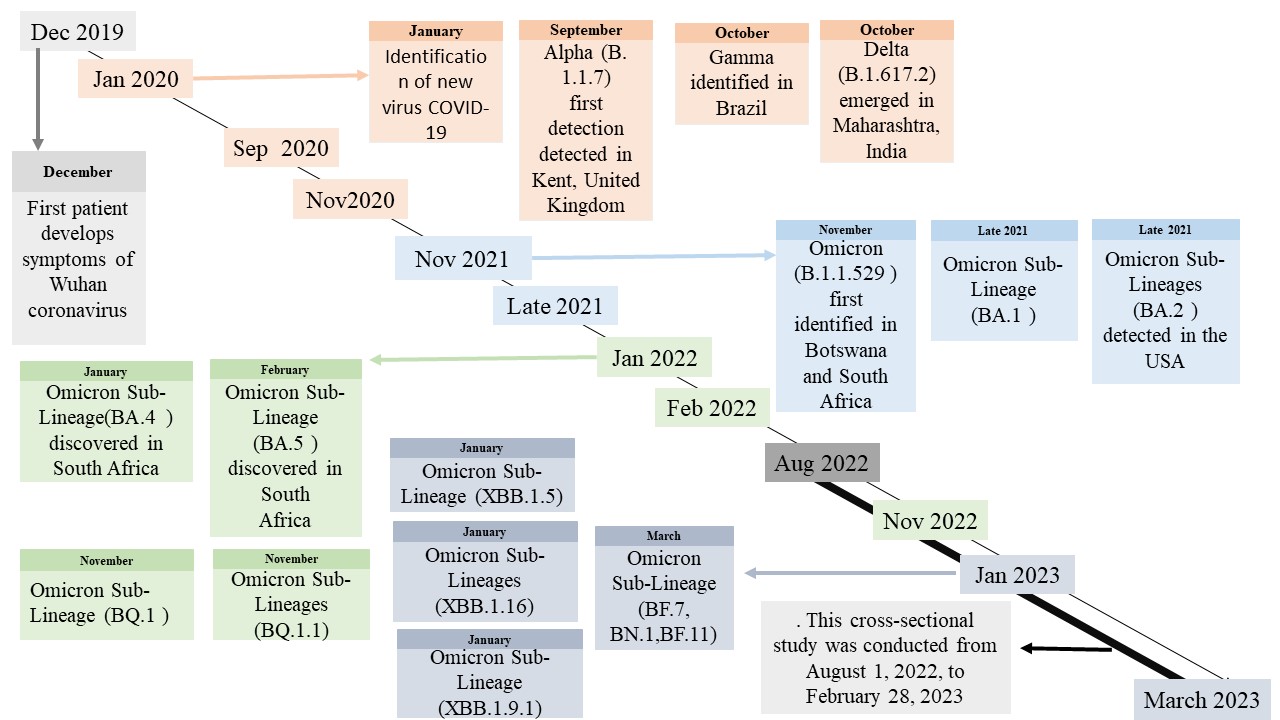


Supplement Figure 1

Timeline of different variants of concerns for SARS-CoV-2

Note:

The figure presents a comprehensive timeline depicting the emergence of various variants. This timeline spans from the virus's origin in 2019 to the most recent variant of concern discovered in early March 2023. The current cross-sectional study was conducted from August 1, 2022, to February 28, 2023 (represented by thick black line)


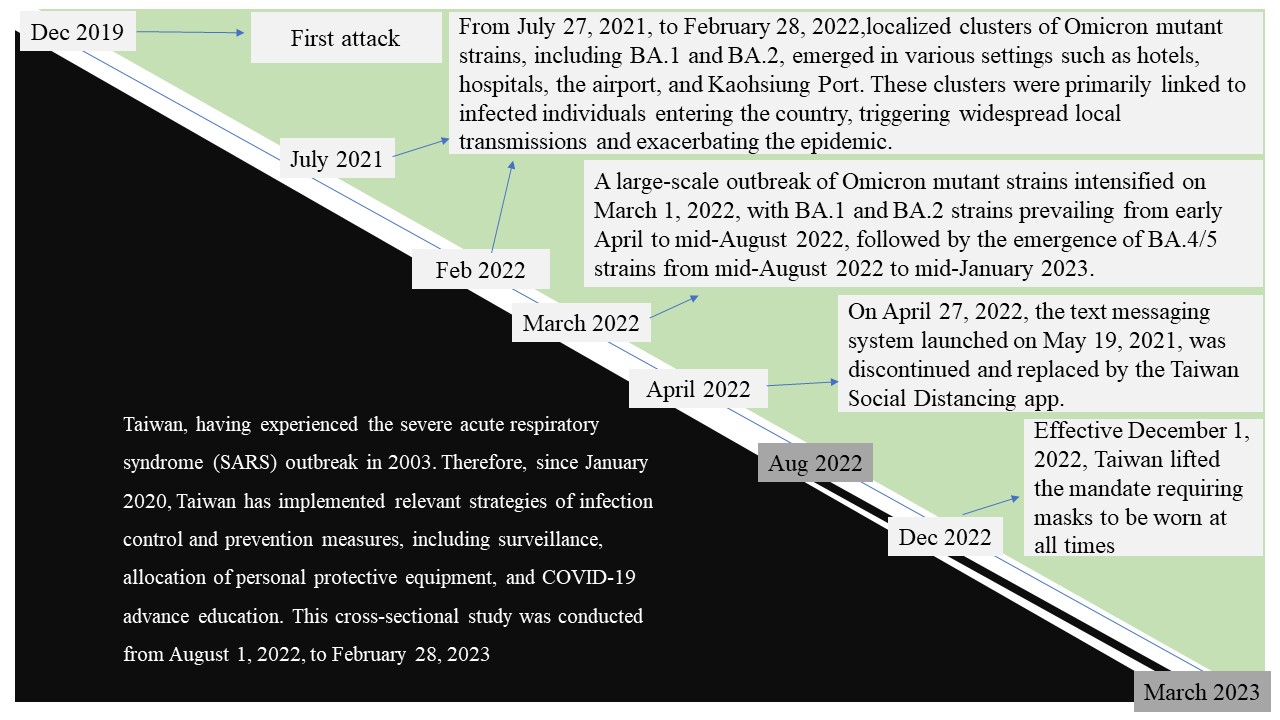


Supplement Figure 2

Timeline of Taiwan's COVID-19 infection prevention and control policies from 2021 to March 2023

Note:

This figure outlines the timeline of policy adjustments, distinguishing between the hospital's policy (represented by the black background) and the community's policy (denoted by the green background). The current cross-sectional study was conducted from August 1, 2022, to February 28, 2023 (represented by thick black line).
